# Assessing the Contribution of the Environmental Parameters to Eutrophication with the Use of the “PaD” and “PaD2” Methods in a Hypereutrophic Lake

**DOI:** 10.3390/ijerph13080764

**Published:** 2016-07-28

**Authors:** Ekaterini Hadjisolomou, Konstantinos Stefanidis, George Papatheodorou, Evanthia Papastergiadou

**Affiliations:** 1 Laboratory of Marine Geology and Physical Oceanography, Department of Geology, Patras University, Patras 26504, Greece; George.Papatheodorou@upatras.gr; 2 Department of Biology, University of Patras-University Campus Rio, Patras 26500, Greece; kstefani@chi.civil.ntua.gr (K.S.); evapap@upatras.gr (E.P.); 3 Sector of Water Resources and Environmental Engineering, School of Civil Engineering, National Technical University of Athens, Athens 15780, Greece

**Keywords:** Artificial Neural Network, eutrophication, lake, environmental parameter, “PaD” method, “PaD2” method

## Abstract

Lake Pamvotis (Greece) is a shallow hypereutrophic lake with a natural tendency to eutrophication. Several restoration measures were applied, but with no long-term success. To examine the causes for this an Artificial Neural Network (ANN) was created in order to simulate the chlorophyll-*a* (Chl-*a*) levels and to investigate the role of the associated environmental parameters. The ANN managed to simulate with good correlation the simulated Chl-*a* and can be considered as a reliable predictor. The relative importance of the environmental parameters to the simulated Chl-*a* was calculated with the use of the “Partial Derivatives” (“PaD”) sensitivity method. The water temperature (WT) and soluble reactive phosphorus (SRP) had the highest relative importance, with values of 50% and 17%, respectively. The synergistic effect of the paired parameters was calculated with the use of the “PaD2” algorithm. The SRP-WT paired parameter was the most influential, with a relative contribution of 22%. The ANN showed that Lake Pamvotis is prone to suffer the effects of climatic change, because of the major contribution of WT. The ANN also revealed that combined nutrients reduction would improve water quality status. The ANN findings can act as an advisory tool regarding any restoration efforts.

## 1. Introduction

Over the past few decades eutrophication has emerged as a serious problem affecting the water quality of many lakes worldwide, mainly as a result of increased nutrient loadings related to human activities [1]. Eutrophication is related with phytoplankton overproduction and has as a common proxy: the chlorophyll-*a* (Chl-*a*) parameter. Eutrophic conditions in a water column may lead to a Harmful Algal Bloom (HAB) event [2]. Three different categories of HABs can be distinguished: toxic algae; potentially toxic algae; and high-biomass blooms, which are called “red tides” [3].

In the case of toxic HABs events, toxin-producing algal species are involved. In freshwater lakes most of these toxic species are cyanobacteria, formally called blue-green algae [4]. Because of their toxin production, cyanobacteria have been characterized as potential key hazardous pollutants, by the European Water Framework Directive (2000) (2000/60/EC) [5]. Aquatic organisms may suffer from poisoning because of cyanotoxins released from cyanobacteria when a HAB occurs [6]. The role of cyanotoxins in recreational waters and their effect on human health have started to be under examination the recent years [7]. It is reported that about 60,000 intoxication incidents, with an overall mortality rate 1.5%, take place per year globally because of algal toxins [8]. 

High biomass blooms are associated with hypoxia and anoxia, with partial or even total oxygen depletion in the water [9]. In hypoxic waters aquatic ecosystems and public health is under serious threat. Besides the negative impact on public health and aquatic organisms, high biomass blooms are responsible for a series of other problems affecting water quality, like bad odor and algal scum formation [10]. The economic cost of these eutrophication related problems is huge. As it is stated by Dodds et al. [11] the economic cost of eutrophication in U.S. freshwaters reached $2.2 billion annually, with the greatest economic loses related with lakefront property values and recreational use. 

Understanding the links between Chl-*a* and the associated environmental parameters is the first critical step for eutrophication management. The linkage between eutrophication and environmental parameters is often non-linear and complex [12], so the simple linear structure of a regression model cannot be applied and other mathematical methods are more suitable for lake modelling. Artificial Neural Networks (ANNs) have proven to be a powerful tool in lake eutrophication assessment [13], because of their ability to model phenomena involving non-linear and complex data [14].

The aim of this study was the creation of an ANN model that would simulate the Chl-*a* and the examination of the impact of each environmental parameter associated with eutrophication. The necessity for evaluating in detail the role of each environmental parameter arose because despite several restoration attempts, Lake Pamvotis remains a hypereutrophic lake. The contribution of each parameter was calculated with the use of the Partial Derivatives (“PaD”) method. The coupled effect of the parameters were calculated with the use of the “PaD2” method, in an attempt to have a more thorough look into eutrophication process and to examine the synergistic effects between the environmental parameters. To our knowledge this is the first modelling study where the “PaD2” algorithm is applied in order to examine the synergistic effect of environmental parameters on the simulated Chl-*a*.

The “Pad2” algorithm is a two-way interaction sensitivity method designed for ANNs that provides information regarding the impact of the several combinations of input parameters to the modeled output. In that way the synergistic effects of the environmental parameters is examined and useful conclusions regarding the trophic production can be extracted. By applying the “Pad2” algorithm the input environmental parameters synergy mechanism is revealed and the environmental parameters’ interactions are evaluated by the ANN model. Therefore this ANN modelling study can be considered an advanced management tool compared with other ANN modelling studies that examine only the one-way sensitivity of environmental parameters. The application of these two ANN model sensitivity methods produced interesting results that can act as an advisory tool for any future restoration attempts. 

## 2. Materials and Methods

### 2.1. Study Area and Data Collection

**Figure 1:**
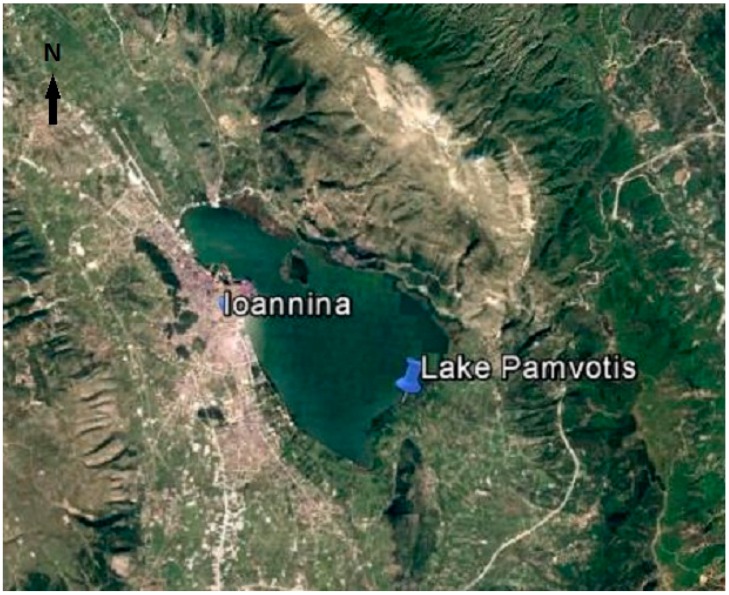
Satellite map of the study area, where Ioannina city is located northwestern. The urban area of Ioannina city is observed in the west side of the lake.

Lake Pamvotis is a shallow Mediterranean lake located in northwestern Greece (Figure 1) with mean depth 4.3 m and maximum depth 7.5 m, raising about 470 m above sea level [15]. It occupies an area of about 22.8 km^2^ and is located next to the city of Ioannina. Pamvotis Lake has been listed among the Natural Special Conservation Areas under the European Community Council Directive on the conservation of natural habitats and of wild fauna and flora (Habitats Directive, European Community, 92/43). 

Lake Pamvotis can be consider as a “closed” hydrological system, as it has no natural surface outflows and is recharged by karstic springs [15,16]. During the last several decades the lake has become more eutrophic due to impacts from agricultural activities, livestock, nearby industrial units, discharges of domestic sewage, irrigation, sediment deposit and introduction of alien fishes [17]. 

A detailed overview of the key changes in the trophic state of the Lake Pamvotis can be found in the articles of Papatheodorou et al. [15]; Kagalou et al. [16]; Papastergiadou et al. [17] and a summary of their recorded data is given below. The nutrient and Chl-*a* concentrations, presented for three different periods from 1985 to 2005, indicate an eutrophic-hypertrophic profile of the trophic state of the lake. Specifically, during the 1985–1988 period the mean annual Chl-*a* concentration was 76.91 mg/m^3^, demonstrating a highly eutrophic to hypertrophic classification of the trophic state. After the sewage diversion that occurred in early 1990s the annual mean of Chl-*a* decreased to 21.21 mg/m^3^, still showing strong signs of eutrophication. However, from samplings carried out in 2004–2005 it was demonstrated that Chl-*a* levels increased significantly, with an annual mean of 79.23 mg/m^3^ suggesting a shift of the trophic state towards hypertrophic conditions. 

In previous years and particularly in 2005, massive cyanobacterial surface blooms were recorded from early summer to late autumn [16]. More recently, Gkelis et al. [18] assessed the occurrence of cyanobacteria blooms in Lake Pamvotis and highlighted the co-occurrence of more than one cyanotoxins during the warm period and the potential risk for human health. These findings clearly suggest that the trophic status of the lake remains highly eutrophic despite the management practices that have been applied the last decades.

In an attempt to improve the lake trophic status, in 1986 Lake Pamvotis was stocked with several native fish species and the exotic planktivorus *Ctenopharygodon idella*. The result of this intervention is debatable, since it led to great decline of submerged vegetation [19,20]. In 1992 the operation of the sewage treatment unit and the consequent discharge of the effluents into Lapsista channel began [17].

The water quality parameters were collected during the monitoring period from June 2004–August 2005 on a monthly basis. Eleven monitoring stations inside Lake Pamvotis were used for the data collection. The measured parameters were SRP, DIN species (nitrite, nitrate, ammonium), carbonate (CO_3_), bicarbonate (HCO_3_), pH, electric conductivity (EC), water temperature (WT), dissolved oxygen (DO), Secchi disk (SD) and chlorophyll *a* (Chl-*a*). 

### 2.2. ANNs Methodologies

Multi-layer feedforward ANNs are a very popular category of ANNs, mainly because they are suitable for function approximation. Feedforward ANNs have acyclic topology with no feedback loops among their layers and are used for approximating non-linear mappings among inputs and outputs [21]. A feedforward ANN has at least three layers and each layer consists of neurons. The first layer is the input layer that imports the input parameters to the network, one or more hidden layers and the output layer that gives the final result. For every neuron there is synaptic weight that connects it with every neuron of the next layer. The weighted sum of a neuron’s inputs produces an output with the use of a transfer function [22]. The relationship that gives the ANN output from the *j*th neuron (*oj*) as given by Kazemi Yazdi and Scholz [23] has the form:

(1) $$o_{j}=f(u_{j})$$

and:

(2) $$u_{j}=\sum w_{ij}x_{i}+z_{j}$$

where *f* is a transfer function, *x_i_* is the input from it neuron belonging to the immediate previous layer, *w_ij_* is the synaptic weight that connects *x_i_* with the *j*th neuron and *z_j_* a bias term. The most widely used transfer factions are the linear transfer function, the log-sigmoid transfer function and the tangential sigmoid transfer function. ANNs are trained with the use of a learning algorithm. Feedforward ANNs are usually trained with the use of a back-propagation algorithm. During the learning procedure the synaptic weights are adjusted so they minimize an error function, usually the mean square difference between the predicted and the given output [24,25].

The number of hidden neurons has a major role in the good performance of an ANN. With a small number of hidden neurons the ANN will not be able to produce good results. Meanwhile for a big number of hidden neurons the phenomenon of overfitting occurs [26]. Overfitting exists when the error for the training set is small, but the ANN has large errors for new data. In that case the ANN hasn’t learned to generalize to new situations [27]. To avoid overfitting many empirical rules that suggest the maximum number of hidden neurons have been proposed. One such practical rule is the one proposed by Maier et al. [28]:

(3) $$N^{H}\leq 2N^{I}+1$$

and:

(4) $$N^{H}\leq 2N^{TR}/(N^{I}+1)$$

where *N^H^* is the number of hidden layer neurons, *N^I^* the number of inputs and *N^TR^* the number of training samples. The maximum *N^H^* must be the smallest number calculated by those two equations. The determination of the optimal number of hidden neurons and layers is decided after a trial and error procedure [29]. In order to prevent an ANN’s overtraining early stopping and regularization methods are used. In early stopping the data set is divided into three subsets: training set, validation set and test set. The network training stops when the errors for the validation set begin to rise, indicating that the network had begun to overfit the data [30]. The regularization method involves modification of the network performance function that is the sum of squares of the network errors in the training set. By using the modified performance function will cause the network to have smaller weights and biases, and this will force the network response to be smoother and less likely to overfit [27]. 

The Partial Derivatives (“PaD”) method, presented by Dimopoulos et al. [31], is a technique for measuring the ANN’s sensitivity regarding its input parameters and is a single parameter interaction method. This method has two parts. As analyzed by Gevrey et al. [32] the partial derivatives of the output for small changes of each input are computed and then the relative contribution of each input variable is classified. The sensitivity of the ANN’s output for the input variable *xi* is symbolized as *SSDi* and according to Dimopoulos et al. [31] is the sum of the squared partial derivatives obtained per input variable and has the following form:

(5) $$d_{ji}=S_{j}\sum_{h=1}^{nh}w_{ho}I_{hj}\left(1-I_{hj}\right)w_{ih}$$

where *N* is the observations number of the data set; *d_ji_* the partial derivatives of the output *y_j_* with respect to input *x_j_*. The equation for *SSDi* is given below:

(6) $$SSD_{i}=\sum_{j=1}^{N}{\left(d_{ji}\right)}^{2}$$

where *nh* the neurons number of the hidden layer; *I_hj_* the output of the *h*-th hidden neuron for the *j*-th input; *w_ih_* the weight connecting the *i*-th input neuron and the *h*-th hidden neuron; *w_ho_* the weight connecting the output and the *h*-th hidden neuron; *S_j_* is the derivative of the output *y_j_* with respect to input *x_j_*. 

The “PaD2” method examines the synergistic effects between the input parameters and is considered a two-way interaction sensitivity analysis method. The application of the “PaD2” method is very similar with the application of the “PaD” method. According to Gevrey et al. [33] the first part of the “PaD2” method that gives the partial derivatives of the output *y_j_* with respect to inputs *x_ji_* and *x_j_*_2_ is given by the following equation:

(7) $$\begin{array}{c} d_{ji}=S_{j}[s_{j}\sum_{h=1}^{nh}w_{1h}w_{ho}I_{hj}\left(1-I_{hj}\right)\sum_{h=1}^{nh}w_{2h}w_{ho}I_{hj}\left(1-I_{hj}\right)\\ +\sum_{h=1}^{nh}w_{1h}w_{2h}w_{ho}I_{hj}\left(1-I_{hj}\right)\left(1-2I_{hj}\right)] \end{array}$$

where the symbols were explained above, except *s_j_* that is the second derivative of the output neuron with respect to its input [31,34]. The relative contribution of the coupled variables to the ANN is the sum of squared partial derivatives of the coupled variables and is calculated as below:

(8) $$SSD_{12}=\sum_{j=1}^{N}{\left(d_{j12}\right)}^{2}$$

### 2.3. ANN Model Development

Using Principal Components Analysis (PCA) the selection of ANN’s input parameters is done [35,36]. PCA is often combined with ANN modelling, because by that way dimension reduction is enabled and the model’s computational complexity is reduced, so the possibility of a model’s misconvergence and poor accuracy is eliminated [37]. Based on PCA results the carbonate and bicarbonate parameters were excluded, since they contribute less than 2% to the total variation of the data set. The resulting input parameters are SRP, DIN, pH, EC, WT, DO and SD and the model output is Chl-*a*. In eutrophication modelling with ANNs it is a common practice the Chl-*a* to be log transformed and then the data set to be normalized [38], as in that way the network performance is improved. After network training the data is set back to the initial form. 

The method of regularization was used for avoiding model’s overfitting, for the regularization method the data set is divided into training set and test set. Therefore the data set consisting of 161 samples was divided into two subsets. The training set containing 80% of the data and the test set 20%. The ANN’s training algorithm was the Levenberg-Marquardt (LM) algorithm, because the LM algorithm has fast learning speed and is the best training algorithm among the rest variations of back-propagation algorithms for medium-sized networks [36,39]. The sigmoid transfer function having the form:

(9) $$f\left(x\right)=\frac{1}{1+e^{-x}}$$

was used as the activation function between the layers and by that way the non-linearity was imported into the model. 

With the use of MatLab software, the ANN was simulated several times with different numbers of neurons in the hidden layer to find the optimal number of neurons as proposed by Sener et al. [40], always keeping in mind the restrictions of the maximum number of neurons as given by the Equations (3) and (4). The optimal neurons number was 10 neurons for the hidden layer, seven neurons for the input layer and one neuron for the output layer. The resulting ANN optimal topology is thus 7-10-1, based in the form L1-H1-L2, where L1 is the number of neurons in the input layer, H1 the number of neurons in the hidden layer and L2 the number of neurons in output layer The best ANN’s model performance was calculated for the test set using the coefficient of determination (*R*^2^) and the Absolute Relative Error (RE) since they are commonly used statistical variables for evaluating model performance [41]. The equations that give *R*^2^ and RE are given respectively as below:

(10) $$R^{2}={\left[\frac{\sum \left(o_{i}-\bar{o_{i}}\right)\left(s-\bar{s_{i}}\right)}{\sqrt{\sum {\left(o_{i}-\bar{o_{i}}\right)}^{2}\sum {\left(s-\bar{s_{i}}\right)}^{2}}}\right]}^{2}$$

(11) $$\text{RE}=\frac{1}{N}\sum_{i=1}^{N}\frac{\left|s_{i}-\bar{o_{i}}\right|}{o_{i}}$$

where *o_i_* is the observed value; *o_i_* is the average observed value; *s_i_* the simulated value; *s_i_* the average simulated value and *N* the observations number. The ANN model performance results for all the data subsets are presented in Table 1.

## 3. Results and Discussion

### 3.1. ANN’s Simulation Results

**Figure 2:**
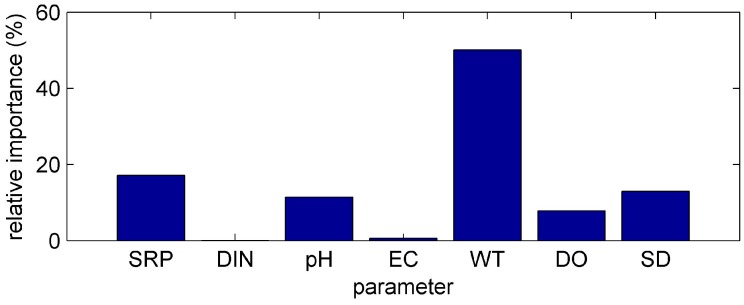
Relative contribution of the input parameters to the ANN model with the use of Partial Derivatives (“PaD”) sensitivity method.

One-way sensitivity analysis was carried out in order to examine how strong the effect of each input parameter on the simulated output is. The “PaD” method was preferred among other ANN sensitivity techniques, since the “PaD” method is the most stable [31]. The relative importance of input variables to the ANN was found to have the following order of contribution according the results of the “PaD” method: WT, SRP, SD, pH, DO, EC, DIN (Figure 2). The “PaD” method has the ability to discriminate the input variables into minor and major contributing environmental parameters [42]. In our case the input parameters WT, SRP, SD, pH, DO have a major relative importance, while the input parameters EC, DIN have minor relative importance to the simulated Chl-*a.*

**Figure 3:**
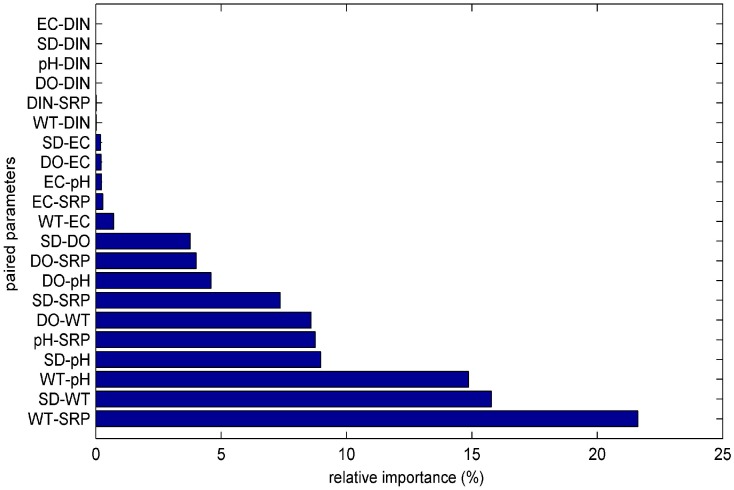
Relative importance of the paired input parameters to the ANN with the use of “PaD2” sensitivity method.

The “PaD2” algorithm was used to calculate the synergistic effect of paired input parameters on the modeled Chl-*a.* For that purpose 21 combinations of paired input parameters were created. The evaluation of the impact for a small change of these paired parameters to the ANN was done with the application of the “PaD2” algorithm. The relative contribution of these interactions are given in Figure 3. 

**Figure 4:**
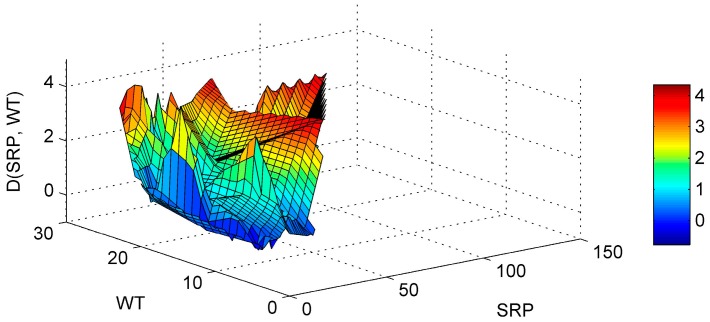
Partial Derivative (D) of the ANN response to the paired interaction of the soluble reactive phosphorus (SRP) and water temperature (WT) variables.

The sum of the five most influential paired parameters (WT-SRP, SD-WT, WT-pH, SD-pH, pH-SRP) described up to 70% of the ANN model sensitivity. The graphical representation of the most influential parameters enables us to produce useful results regarding the parameters’ interactions. The WT-SRP (Figure 4) is the most influential interaction and has a relative importance of almost 22%. As it is graphically observed the Chl-*a* gradients start to rise for high SRP concentrations combined with high WT. Meanwhile the same high WT values combined with lower SRP levels reveal that Chl-*a* gradients have the tendency to decrease. Therefore it is obvious that a decrease in SRP levels would lead to a decrease to Chl-*a* values, even during the hot months that are associated with high algal production [43].

**Figure 5:**
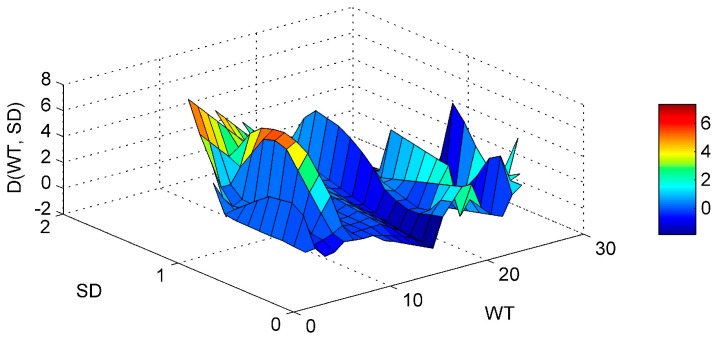
Partial Derivative (D) of the ANN response to the paired interaction of the secchi disk (SD) and water temperature (WT) variables.

**Figure 6:**
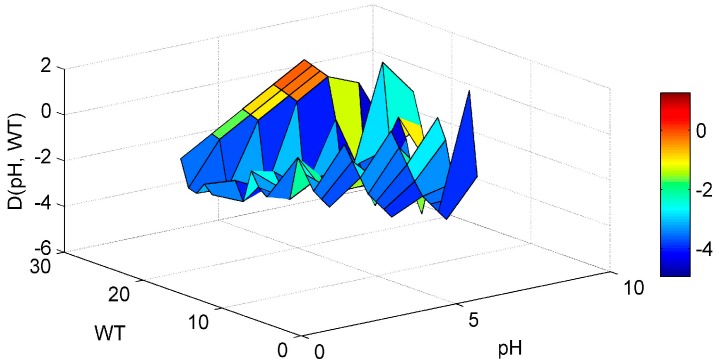
Partial Derivative (D) of the ANN response to the paired interaction of the pH and water temperature (WT) variables.

**Figure 7:**
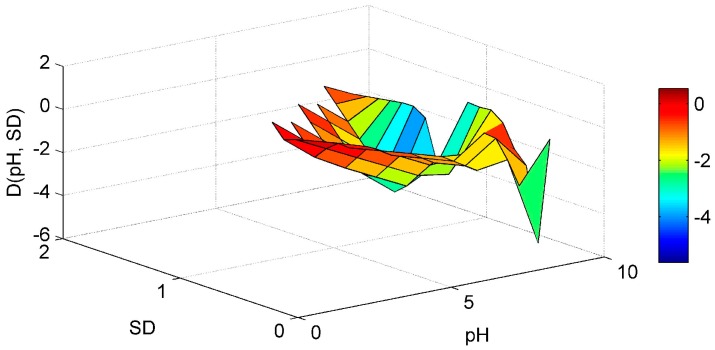
Partial Derivative (D) of the ANN response to the paired interaction of the pH and secchi disk (SD) variables.

**Figure 8:**
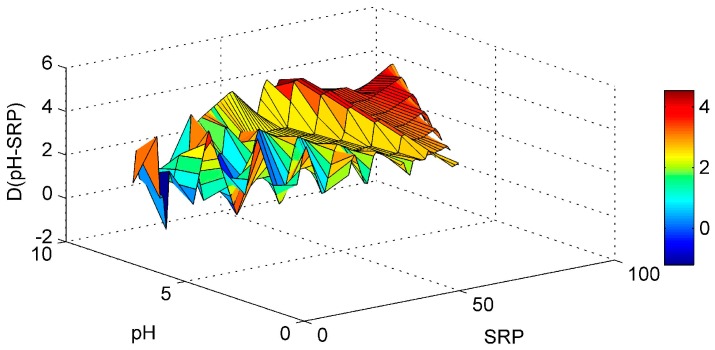
Partial Derivative (D) of the ANN response to the paired interaction of the pH and soluble reactive phosphorus (SRP) variables.

Regarding the SD-WT interaction (Figure 5) it is noticed that the Chl-*a* derivative starts to rise in an area of low WT values and medium-high SD values, but no clear conclusions can be reached. The ANN model managed to interpolate well the WT-pH interaction (Figure 6). For elevated pH and high WT there is a tendency for the Chl-*a* gradient to rise. In that way the high pH levels observed related with increased algal activity and high WT that stimulates algal production, are associated. This is in agreement with a study of Trolle et al. [44] which noted that high Chl-*a* values are linked with high pH, especially during summer. The SD-pH combination (Figure 7) gives negative Chl-*a* derivatives. This behavior can be explained by the fact that small increase of SD values due to application of the “PaD2” algorithm results in contrary behavior regarding Chl-*a* production. The SD is negatively correlated with Chl-*a* levels [45]. The pH-SRP combination (Figure 8) although it follows no specific pattern, shows that for high SRP and pH levels the Chl-*a* gradients take their highest values and for lower SRP levels the derivatives have smaller values, so the ANN managed to correctly connect the SRP effect on Chl-*a* and to associate it with high pH because of increased algal production. 

**Figure 9:**
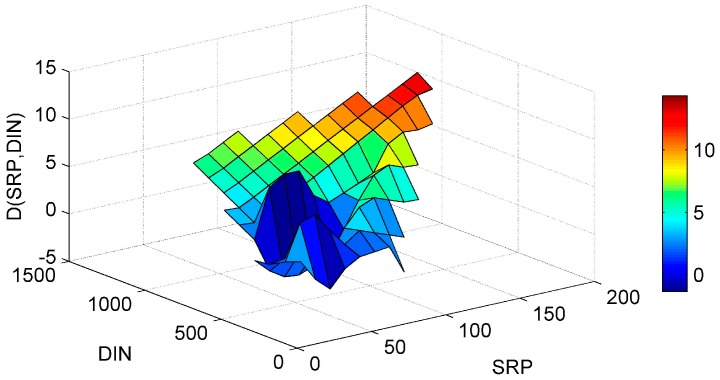
Partial Derivative (D) of the ANN response to the paired interaction of the soluble reactive phosphorus (SRP) and dissolved inorganic nitrogen (DIN) variables.

The SRP-DIN paired parameter was calculated to have no major relative importance on the simulated Chl-*a.* Despite this finding the impact of DIN on Chl-*a* production should not be underestimated, because DIN high levels are associated with continued serious eutrophication problems caused by non-N_2_ fixing cyanobacterial blooms [46]. Additionally all the ANN simulations without the DIN as an input parameter had worse performance than the ANN simulations with the DIN as an input parameter, verifying the importance of DIN on the Chl-*a* in a mathematical way. For these reasons a graphical representation of the SRP-DIN (Figure 9) interaction is also presented, in order to exam the synergistic effect on Chl-*a* behavior. 

The synergistic effect of the SRP-DIN follows a clear pattern regarding the Chl-*a* gradient. For high SRP and DIN values the Chl-*a* derivative has the bigger increase and as the SRP and DIN values decrease the Chl-*a* derivative tends to decrease accordingly. The reduction of DIN or SRP gives a reduction for Chl-*a* and the combined reduction of nutrients yields to even higher reduction [47], something that is verified by the “PaD2” algorithm findings. 

### 3.2. Implications for Management and Restoration

This ANN modelling study was aimed at evaluating the influence of each environmental parameter on the simulated Chl-*a.* A need for careful examination of the environmental parameters resulted, since it was observed that despite several restoration measures that were applied the last decades to Lake Pamvotis, no lasting water quality improvement was achieved. The impact magnitude of the parameters and the way that they interact with other parameters regarding algal production needed to be investigated. For that reason the “PaD” and the“PaD2” algorithms were used, producing interesting findings about the parameters.

An ANN model was chosen for this modelling study instead of any other modelling technique because of the ANNs’ ability to model complex systems, making them ideal for lake modelling. The trophic function of a lake can be modeled with the use of an ANN usually with a good correlation, as in this modelling study. Therefore the created ANN can be considered as a reliable predictor of Chl-*a* providing mathematical trustworthy relationships between the modeled Chl-*a* and the environmental parameters. Sensitivity analysis algorithms are the linkages between the Chl-*a* and the environmental parameters’ interactions. Furthermore the“PaD2” method enables us to assess the synergism of coupled parameters on the simulated Chl-*a*, providing us an advanced management tool comparing with other ANN modelling studies that deal only with one-way parameter sensitivity analysis. Interesting trends between variables synergistic effect and the simulated Chl-*a* were revealed, like the WT synergism with the SRP that makes restoration efforts more difficult. 

Some of the input parameters were found to be more influential than others; however the importance of each input parameter must not be underestimated. All the ANN model's input parameters were found to have an effect on the simulated Chl-*a* and an omission of any of the input parameters as an input would lead to poor ANN model performance. For that reason it was decided to pay attention to the DIN as well, even though it was found to have small relative importance for the Chl-*a.* The increased WT related with climatic change increases the risk for a potentially toxic cyanobacterial bloom in lakes with high nitrogen concentration [48]. The high relative importance of the SRP revealed that it is the key nutrient for Lake Pamvotis and has a significant role in the algal production. In a relevant study of Lake Pamvotis Papatheodorou et al. [15] also found that the key nutrient is the SRP. The combined nutrients “synergistic” effect was pronounced in summer and fall, when algal production is highest [45]. For that reason lake modelling studies that examine the nutrients reduction effect were carried out, like the one of Mateus et al. [49]. Our modelling study confirms these results, since the paired SRP-DIN parameter showed that a decrease of nutrients would lead to a Chl-*a* decrease. The “PaD2” algorithm findings regarding the nutrients behavior could act as a practical management guide tool for decision makers.

Another interesting finding was that the most important parameter was the WT, confirming that way Kagalou et al. [16] theory that the effects of climate change for Lake Pamvotis may be greater than those caused by anthropogenic pressure. It is generally accepted that temperature increase leads to more intense eutrophication symptoms [50,51]. Keeping in mind the results of the SRP-DIN synergistic effect and the major role of WT, then the necessity for extra nutrient reduction is obvious because the more eutrophic a lake is then the effect of temperature is greater on algal production [52]. As it is stated by Papastergiadou et al. [17] Lake Pamvotis is unlikely to switch to clear water conditions and has a tendency for eutrophic conditions, but improvement of water quality can be achieved through specific management practices like nutrient reduction from the catchment area and the sediment, increasing the flushing rate by increasing the karstic springs’ discharge by rediverting the springs located on the northern shore into the lake, and by biomanipulation practices to minimize the population of the allochthonous benthivorous species. The Lake Pamvotis natural tendency for eutrophication can be partly explained by the paired SRP-WT parameter computational results. Climatic change favors the phosphate release from anoxic sediments and increases phosphate internal loading, especially during hot months [53]. Prolonged and intensified eutrophication might be related with an internal nutrient supply in the water body, even if external input no longer exists [54]. The climatic change and the associated WT increase must be taken into consideration for any restoration measures, since Lake Pamvotis is highly susceptible to increased WT effects.

For Lake Pamvotis other environmental factors that affect eutrophication should also be examined. For example modelling studies that examine the role of fish, macrophytes and zooplankton in lake restoration have been initiated. Models dealing with other restoration techniques like biomanipulation could be developed in order to provide alternative eutrophication management options. Sagehashi et al. [55] created such a model for a shallow basin that combines as parameters planktivorous fish, three types of zooplankton, two types of algae and nutrients. Based on that model they simulated some restoration methods like biomanipulation, dredging and nutrient loading reduction and the analog effect on water quality. It is suggested as this ANN modelling study of Lake Pamvotis should be updated with new extra environmental parameters that would include long-term monitored data like the ones mentioned above, e.g., planktivorous fish and zooplankton density.

## 4. Conclusions

Lake algal production is considered a very complex issue and the relationships with the associated environmental parameters are difficult to examine. However ANNs are ideal for lake modelling because of their ability to model non-linear situations. A small change of an input variable produces the sensitivity result of the ANN and the way the modeled output responds to that change. With the use of the “PaD” and “PaD2” methods the one-way sensitivity and the two-way sensitivity are calculated accordingly, revealing in that way how Chl-*a* responds to changes of the environmental variables. The results given by the “PaD” and “PaD2” methods are very important for understanding a lake function and how each environmental variable affects the algal production. Especially for eutrophic and hypertrophic lakes, these findings have the role of advisory restoration tools. For example by using the “PaD” method the key nutrient is found. Also with the use of “PaD2” method it is examined if the restoration measures must follow a one nutrient control policy or combined nutrient reduction. Meanwhile the synergistic effect between the environmental parameters can act as a warning tool for hypertrophic lakes, since the “PaD2” method can reveal if for the hot months and for certain nutrient levels a HAB event might occur. 

The ANN managed successfully to simulate the Chl-*a* levels with a good correlation so the ANN model can be considered as a reliable predictor, based on which the effect of the environmental parameters can be calculated. The ANN’s one-way sensitivity analysis was performed with the use of “PaD” algorithm. The most contributing parameters were found to be the WT and SRP parameters. The synergistic effect of the parameters was calculated with the use of the “PaD2” algorithm, a paired sensitivity analysis method. The paired parameters interactions revealed that the SRP-WT combination had the higher relative contribution to the simulated Chl-*a.* These results showed that the SRP is the lake’s key nutrient and that the lake algal production is highly affected by climatic change. 

The DIN had no major relative contribution. However the role of DIN must not be underestimated in the restoration attempts, since the ANN results showed that the synergistic effect of SRP-DIN has a clear association pattern with the algal production. Taking all these into consideration, it is indicated that the management measures taken for lake restoration must be updated and new restoration practices should be applied alongside the existing ones. This ANN model combined with the “PaD” and “PaD2” algorithms has become a successful management guiding tool revealing trends between Chl-*a* production and the environmental variables. 

## Figures and Tables

**Table 1:** ANN’s performance for the training, test and whole set based on the RE, Absolute Relative Error (RE) and *R*^2^, coefficient of determination.

Model Performance	*R*^2^	RE
Training set	0.76	0.57
Test set	0.82	0.64
Whole set	0.77	0.59

## Abbreviations

The following abbreviations are used in this manuscript:

SRP	Soluble Reactive Phosphorus
DIN	Dissolved Inorganic Nitrogen
EC	Electrical Conductivity
WT	Water Temperature
DO	Dissolved Oxygen
SD	Secchi Disk
RE	Absolute Relative Error
R^2^	Coefficient of Determination
HAB	Harmful Algal Bloom
